# Effect of seven anti-tuberculosis treatment regimens on sputum microbiome: a retrospective analysis of the HIGHRIF study 2 and PanACEA MAMS-TB clinical trials

**DOI:** 10.1016/S2666-5247(23)00191-X

**Published:** 2023-10-10

**Authors:** Emmanuel Musisi, Adam Wyness, Sahar Eldirdiri, Evelin Dombay, Bariki Mtafya, Nyanda E Ntinginya, Norbert Heinrich, Gibson S Kibiki, Michael Hoelscher, Martin Boeree, Rob Aarnoutse, Stephen H Gillespie, Wilber Sabiiti

**Affiliations:** Division of Infection and Global Health, School of Medicine, University of St Andrews, St Andrews, UK; Division of Infection and Global Health, School of Medicine, University of St Andrews, St Andrews, UK; Scottish Association of Marine Science, Oban, UK; Department of Microbiology, Kettering General Hospital, Kettering, UK; Division of Infection and Global Health, School of Medicine, University of St Andrews, St Andrews, UK; Division of Infection and Global Health, School of Medicine, University of St Andrews, St Andrews, UK; National Institute for Medical Research, Mbeya Medical Research Centre, Mbeya, Tanzania; National Institute for Medical Research, Mbeya Medical Research Centre, Mbeya, Tanzania; Division of Infectious Diseases and Tropical Medicine, University Hospital, University of Munich (LMU), Munich, Germany; Kilimanjaro Clinical Research Institute, Moshi, Tanzania; Africa Research Excellence Fund (AREF), London, UK; Division of Infectious Diseases and Tropical Medicine, University Hospital, University of Munich (LMU), Munich, Germany; Fraunhofer ITMP, Immunology, Infection and Pandemic Research, Munich, Germany; Department of Lung Diseases, Radboud University Medical Centre, Nijmegen, Netherlands; Department of Pharmacy, Radboud University Medical Centre, Nijmegen, Netherlands; Division of Infection and Global Health, School of Medicine, University of St Andrews, St Andrews, UK; Division of Infection and Global Health, School of Medicine, University of St Andrews, St Andrews, UK

## Abstract

**Background:**

Respiratory tract microbiota has been described as the gatekeeper for respiratory health. We aimed to assess the impact of standard-of-care and experimental anti-tuberculosis treatment regimens on the respiratory microbiome and implications for treatment outcomes.

**Methods:**

In this retrospective study, we analysed the sputum microbiome of participants with tuberculosis treated with six experimental regimens versus standard-of-care who were part of the HIGHRIF study 2 (NCT00760149) and PanACEA MAMS-TB (NCT01785186) clinical trials across a 3-month treatment follow-up period. Samples were from participants in Mbeya, Kilimanjaro, Bagamoyo, and Dar es Salaam, Tanzania. Experimental regimens were composed of different combinations of rifampicin (R), isoniazid (H), pyrazinamide (Z), ethambutol (E), moxifloxacin (M), and a new drug, SQ109 (Q). Reverse transcription was used to create complementary DNA for each participant’s total sputum RNA and the V3-V4 region of the 16S rRNA gene was sequenced using the Illumina metagenomic technique. Qiime was used to analyse the amplicon sequence variants and estimate alpha diversity. Descriptive statistics were applied to assess differences in alpha diversity pre-treatment and post-treatment initiation and the effect of each treatment regimen.

**Findings:**

Sequence data were obtained from 397 pre-treatment and post-treatment samples taken between Sept 26, 2008, and June 30, 2015, across seven treatment regimens. Pre-treatment microbiome (206 genera) was dominated by Firmicutes (2860 [44%] of 6500 amplicon sequence variants [ASVs]) at the phylum level and *Streptococcus* (2340 [36%] ASVs) at the genus level. Two regimens had a significant depressing effect on the microbiome after 2 weeks of treatment, HR_20mg/kg_ZM (Shannon diversity index p=0·0041) and HR_35mg/kg_ZE (p=0·027). Gram-negative bacteria were the most sensitive to bactericidal activity of treatment with the highest number of species suppressed being under the moxifloxacin regimen. By week 12 after treatment initiation, microbiomes had recovered to pre-treatment level except for the HR_35mg/kg_ZE regimen and for genus *Mycobacterium*, which did not show recovery across all regimens. Tuberculosis culture conversion to negative by week 8 of treatment was associated with clearance of genus *Neisseria*, with a 98% reduction of the pre-treatment level.

**Interpretation:**

HR_20mg/kg_ZM was effective against tuberculosis without limiting microbiome recovery, which implies a shorter efficacious anti-tuberculosis regimen with improved treatment outcomes might be achieved without harming the commensal microbiota.

**Funding:**

European and Developing Countries Clinical Trials Partnership and German Ministry of Education and Research.

## Introduction

Respiratory microbiota has been described as a gatekeeper of respiratory health that modulates host immunity and the resistance to colonisation by pathogens.^1^ Different disease states and exposure to antibiotics have been shown to cause dysbiosis of the microbiome.^2^ It has been shown that treatment with a standard first-line anti-tuberculosis regimen of 2 months of isoniazid, rifampicin, pyrazinamide, and ethambutol (HRZE) followed by 4 months of isoniazid and rifampicin, does not perturb overall microbiome diversity but depletes some immunologically important commensal bacteria, an outcome that might have long-term consequences on an individual’s health.^3^ Short-term tuberculosis regimen courses could shorten the length of microbiome exposure to antibiotics and reduce the risk of long-term damage to the microbiome. Accordingly, novel short-term tuberculosis regimen courses are being investigated, including the 4 month rifapentine–moxifloxacin-containing regimen that was recommended by WHO in 2022.^4^

The human lung is not sterile, even in healthy individuals. The generally diverse core composition of the microbiota in the human lung varies between people in relative abundance and prevalence.^5^ The microbiome can vary based on age (neonates, infants, young people, and adults), diet, and disease.^6^ Diseases such as lung cancer and tuberculosis have been associated with changes in the microbiome characterised by an increase or decrease of some taxa. The lung is the predilection site for *Mycobacterium tuberculosis* infection, and its microbiota are associated with various states of tuberculosis. This means that an intervention, such as antibiotic treatment, adds an extra level of pressure on the microbiome, which can further alter the composition and function of the lung microbiota.^7^ The effect of tuberculosis regimens on lung microbiota is likely to be more associated with antibiotics that have activity against a wide range of Gram-positive and Gram-negative bacteria.^8^ It is thus necessary to investigate the effect of the novel tuberculosis regimen courses, especially those incorporating broad-spectrum high-dose antibiotics.^9–11^

We used sputum to investigate the effect of standard-of-care and investigational tuberculosis regimen courses on the lung microbiota and their implications on treatment outcomes.

## Methods

### Study design

We did a retrospective analysis of the effect of anti-tuberculosis drugs on sputum microbiome using samples that were collected during two clinical trials: PanACEA MAMS-TB (NCT01785186; conducted between May 7, 2013, and March 25, 2014)^12^ and the HIGHRIF study 2 (NCT00760149; conducted between Sept 26, 2008 and Sept 8, 2013).^13^

Sputum samples for the microbiome analysis were obtained from participants in the PanACEA MAMS-TB and HIGHRIF study 2 clinical trials in Tanzania. Approval of the MAMS study was obtained from the Tanzania National Institute for Medical Research (NIMR)-Mbeya Medical Research Centre ethics review committee, the NIMR-National Health Research ethics committee (NatHREC), and University of Munich (study sponsor) ethics committee. HIGHRIF study 2 was approved by the Kilimanjaro Christian Medical College Research Ethics and Review Committee, the Ifakara Health Institute Institutional Review Board, and NatHREC. Both studies were conducted according to Good Clinical Practice guidelines. Participants gave oral informed consent for inclusion in this follow-up analysis.

### Study setting and participants

#### PanACEA MAMS-TB trial

The PanACEA MAMS-TB trial was a five-arm trial assessing four experimental treatment regimens, including isoniazid and 10 mg/kg of rifampicin plus pyrazinamide and ethambutol (HR_10mg/kg_ZE; standard-of-care; isoniazid and 10 mg/kg of rifampicin plus pyrazinamide and SQ109 (HR_10mg/kg_ZQ); isoniazid and 20 mg/kg of rifampicin plus SQ109 (HR_20mg/kg_ZQ); isoniazid and 20 mg/kg of rifampicin plus pyrazinamide and moxifloxacin (HR_20mg/kg_ZM); and isoniazid and 35 mg/kg of rifampicin plus pyrazinamide and ethambutol (HR_35mg/kg_ZE; table 1).

The trial was conducted in three sites in Tanzania and four research institutes affiliated with hospitals in South Africa and is described elsewhere in more detail.^12^ For PanACEA MAMS-TB, only samples from participants in Mbeya, Tanzania, were included in the analysis. All patients who responded well clinically to the treatment or had at least one negative culture towards the end of treatment follow-up were included in this study. Patients were followed up until 6 months after the end of treatment by telephone calls or on-site visits if participants were unwell. The study collected sputum samples taken weekly up to week 12. An outcome of cure, no information, or relapse was assigned at the end of the follow-up. Bacterial load was measured by Mycobacteria growth indicator tube time to positivity and by tuberculosis molecular bacterial load assay (TB-MBLA).^12^

#### HIGHRIF study 2

The HIGHRIF2 study investigated the pharma-cokinetics, tolerability, and bacteriological response of rifampicin administered at varying doses. Details of the study design, sites, and selection of the study participants were published by Aarnoutse and colleagues.^13^ Briefly, the study was conducted between Sept 26, 2008, and Sept 8, 2013, in Kilimanjaro and Dar es Salaam, Tanzania. Participants were given a fixed dose of standard tuberculosis drugs containing rifampicin at 600 mg (HR_600mg_ZE; standard-of-care**)**, 900 mg (HR_900mg_ZE), or 1200 mg (HR_1200mg_ZE) daily (table 1). Study medications were taken together once daily in the morning for 7 days per week. After the intensive treatment phase, all patients were treated according to Tanzanian guidelines for 4 months.

Sputum was collected once per week up to week 12, and at weeks 14, 17, 22, and 26 during the treatment course. Sputum total RNA was extracted as previously described.^14^ To prevent RNA loss after expectoration, sputa from the PanACEA MAMS-TB study were preserved in guanidine thiocyanate containing β-mercaptoethanol, whereas sputa from HIGHRIF study 2 were preserved in Oragene cups (DNA Genotek, Stittsville, ON, Canada). Sputa from both studies were frozen at –80°C until RNA extraction, applying the chloroform–phenol method. RNA extract (4 µL) was used to measure *M tuberculosis* bacillary load by TB-MBLA.

### Procedures

Total RNA was reverse transcribed to complementary DNA (cDNA) using QuantiTect Reverse Transcription Kit (Qiagen, Manchester, UK). The reverse transcription reaction mixture was prepared by adding reverse transcription enzyme (1 µL), transcription buffer (4 µL), and transcription primer mix (1 µL) to the thawed RNA sample before incubation at 95°C for 3 mins. Resulting cDNA was quantified using a high-sensitivity single-stranded DNA Qubit assay (Fisher Scientific UK, Loughborough, UK). The cDNA was diluted with nuclease-free water to ensure the starting template concentration was not too high for PCR.

Primers targeting the V3–V4 region of the 16S rRNA gene were used to amplify the cDNA, as described by Klindworth and colleagues.^15^ Briefly, 2·5 µL of cDNA was added to 17·7 µL of the amplicon PCR master mix containing Taqmix (Qiagen, Manchester, UK; 9·5 µL), forward primer (4 µL), and reverse primer (4 µL). The amplicon PCR conditions were set up as follows: 95°C for 3 min, 35 cycles at 95°C for 30 s, 55°C for 30 s, 72°C for 30 s, and then 72°C for 5 min. The amplicons were cleaned up using AMPure XP reagent (Beckman Coulter, Wycombe, UK) and 10mM tris(hydroxy) methylaminomethane buffer at pH 8·5 and ethanol (appendix p 2).^16^

For library preparation, index PCR was used to label the amplicons using variable DNA adapters following the Illumina metagenomic sequencing protocol. Briefly, 10 µL of cDNA amplicons were added to the index PCR master mix containing KAPA Hifi hot start ready mix (25 µL; Sigma Aldrich, Glasgow, UK) and PCR grade water (10 µL). Index PCR products were cleaned (appendix p 2), quantified using Qubit assay, and then labelled by adding unique indices.

Amplicon concentration was normalised to ensure equal concentration before pooling into one library. Amplicon quality and specificity were assessed using gel electrophoresis with 2·5% agarose gel, SYBRsafe dye (Qiagen, Manchester, UK), and 50 bp ladder. A single band of 600 bp was obtained indicating the specificity of the amplicons in the pooled library (appendix p 2). The concentration of the pooled library was measured and found to be 2·47 ng/µl. Background or cross-contamination was checked by running negative control samples, nuclease-free water, and master mixes for cDNA synthesis and amplicon PCR. These controls were run from in vitro cDNA synthesis to sequencing. The absence of detectable sequences was confirmation of no background contamination.

High-throughput amplicon sequencing was performed at the Integrated Microbiome Resource (Edinburgh Genomics Centre, Edinburgh, UK) on HiSeq 2000 platform. Before sequencing, a bioanalyser quality check confirmed sufficient material for sequencing with no appreciable adapter dimers. Raw reads were filtered, trimmed, and dereplicated, paired reads were merged, denoising was done, and chimaeras were removed using the DADA2 pipeline (1.8) within QIIME2 (version 2020.2). Taxa were allocated to amplicon sequence variants (ASVs) using the SILVA 132 database. Sequences assigned to eukaryotes and archaea were removed. Analyses were performed on the SILVA 132 database for total bacteria and sequences assigned to photosynthetic cyanobacteria were extracted and analysed separately. Alpha diversity metrics (amplicon sequence variant richness [taxa relative abundance], Faith’s phylogenetic diversity [summation of length and number of phylogenetic tree units], Shannon diversity index [number of species scaled by their distribution in the community], and Pielou’s evenness index [distributions of different species in the community]) were calculated for treatment medians within QIIME2 at a rarefaction of 1500 reads after ensuring all samples had reached the rarefaction curve plateau.

### Statistical analysis

We grouped participants based on their region of origin and the difference in their microbiome diversity tested using a Mann-Whitney test. Similarly, we applied a Mann-Whitney test to calculate the difference between pre-treatment and post-treatment alpha diversity under different regimens. We used Spearman’s rank correlation to calculate the correlation between *Mycobacterium* relative abundance and tuberculosis bacterial load measured by TB-MBLA. We applied a one-way ANOVA to test the variation in taxa richness over 8 weeks of treatment among those whose tuberculosis culture was negative, positive, or indeterminate. Indeterminate was defined as a culture in which the tuberculosis status could neither be called positive or negative. All calculations were done in GraphPad Prism (version 9) and statistical significance was accepted at p<0·05.

### Role of the funding source

The funders of the study had no role in study design, data collection, data analysis, data interpretation, or writing of the report.

## Results

Of the 394 samples, 122 (31%) were from HR_600mg_ZE or HR_10mg/kg_ZE, 40 (10%) were from HR_10mg/kg_ZQ, 48 (12%) were from HR_900mg/kg_ZE, 52 (13%) were from HR_1200mg/kg_ZE, 45 (11%) were from HR_35mg/kg_ZE, 42 (11%) were from HR_20mg/kg_ZQ, and 45 (11%) were from HR_20mg/kg_ZM treatment regimens. Demographic data for the participants who provided sputa that were included in the current study are summarised in the appendix (p 2).

A total of 6500 ASVs from individuals treated for tuberculosis were analysed. Pre-treatment taxa were dominated by Firmicutes (2860 [44%]), followed by Bacteroidetes (1170 [18%]), Proteobacteria (845 [13%]), and Actinobacteria (52 [0·8%]). When the microbiome was divided by region of participant origin, Firmicutes remained dominant at 672 (42%; 1600 ASVs) in north–southeast and 2597 (53%; 4900 ASVs) in southwest Tanzania. The proportion of pre-treatment Actinobacteria was higher in north–southeast (304 [19%]) ASVs compared with in southwest (245 [5%]). Bacteroidetes (882 [18%]) and Proteobacteria (735 [15%]) ASVs were proportionally more abundant in southwest than in north–southeast (appendix p 3).

At the genus level, the four most abundant phyla were represented by *Streptococcus* (2340 [36%]), *Neisseria* (650 [10%]), *Veillonella* (390 [6%]), and *Mycobacterium* (325 [5%]) ASVs. When divided by sample origin, *Mycobacterium*, with 208 (13%) ASVs, was the second most dominant genus in north–southeast Tanzania surpassing *Neisseria* and *Veillonella* (appendix p 4).

**Figure 1:**
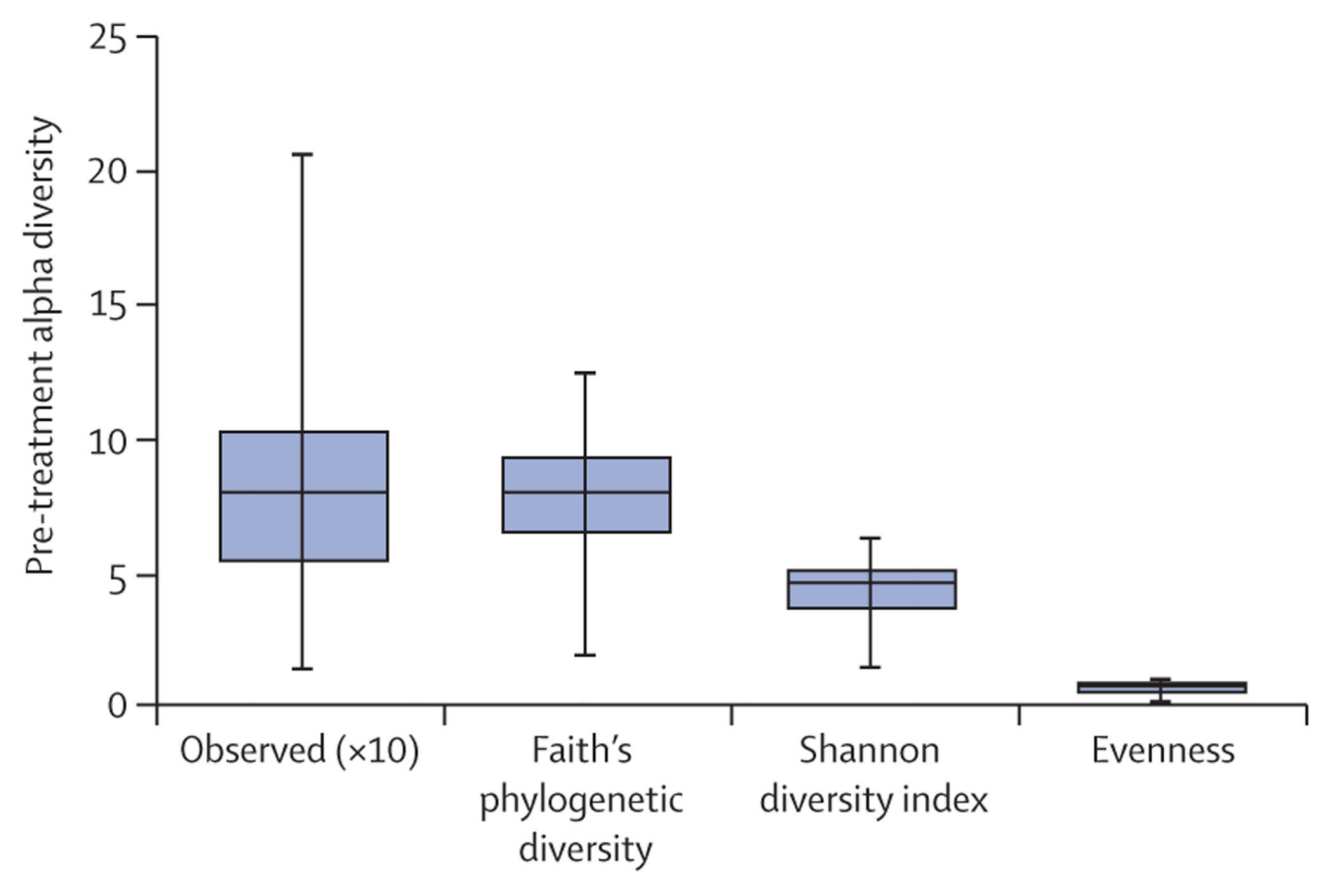
The pre-treatment microbiome richness, distribution, and diversity Some taxa were more represented than others as indicated by low evenness. The scale of observed sequence variants (sample richness) was × 10 of the y-axis value.

Before treatment, the alpha diversity of the whole cohort was median 81 (IQR 17–205) sample richness, 7·9 (2–12) Faith’s phylogenetic diversity, 4·7 (1·6–6·2) Shannon diversity index, and 0·7 (0·3–0·9) Pielou’s evenness. Evenness was comparatively lower than Shannon diversity index and Faith’s phylogenetic diversity, suggesting an unevenly distributed microbiome in which some taxa dominated others (figure 1).

**Figure 2:**
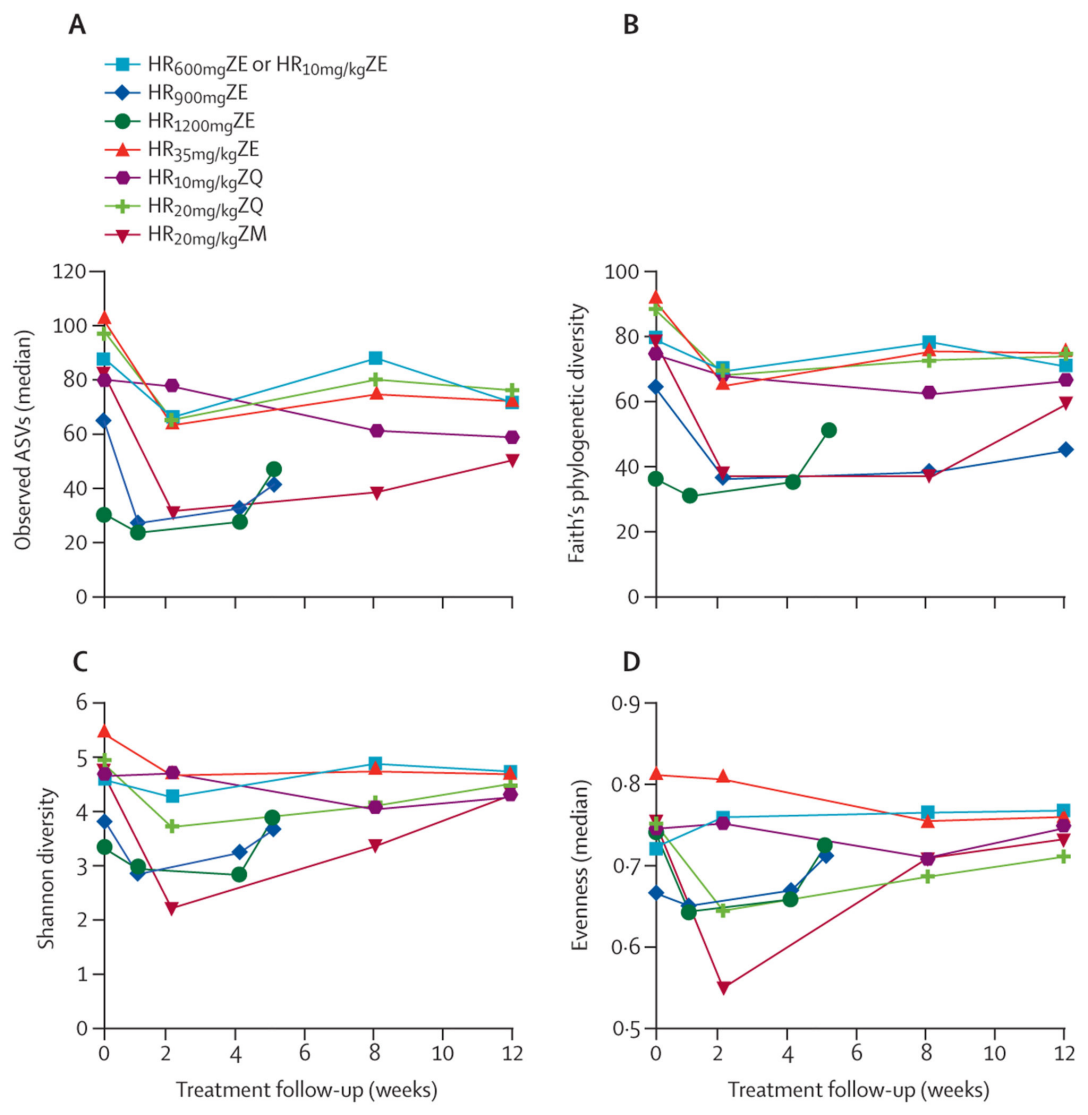
Change in alpha diversity under different regimens following initiation of treatment The highest fall in alpha diversity occurred in the first 2 weeks of treatment, after which alpha diversity began to recover. See table 1 for regimen details. ASV=amplicon sequence variant.

After establishing the pre-treatment alpha and beta diversity, we then explored how this diversity changed under different regimens after the initiation of treatment. In all regimens, the highest reduction in abundance and diversity occurred in the first 2 weeks of treatment. After the first 2 weeks of treatment, abundance and diversity began to recover, achieving pre-treatment level by week 8 after treatment began in most of the regimens. There was a fall-and-rise pattern of alpha diversity induced differently by different regimens. HR_10mg/kg_ZQ was responsible for the smallest reduction of the alpha diversity across all regimens (appendix pp 5–7). HR_20mg/kg_ZM caused the largest depression of evenness, dropping from 0·75 to 0·55. Evenness rose during the HR_10mg/kg_ZE regimen. Overall, the moxifloxacin–rifampicin containing regimen (HR_20mg/kg_ZM), followed by high-dose rifampicin regimen HR_35mg/kg_ZE, caused the largest depression of alpha diversity (figure 2A–D).

Using the Mann–Whitney test, we assessed whether the change in alpha diversity was significantly different between pre-treatment and post-treatment microbiome across the treatment course. Evenness significantly increased in the standard regimen HR_600mg_ZE or HR_10mg/kg_ZE from 0·72 at baseline to 0·80 at week 12 of treatment (p=0·035 at week 2, p=0·024 at week 8, and p=0·034 at week 12 of treatment). Furthermore, the standard regimen did not cause a significant reduction of richness, Faith’s phylogenetic diversity, or Shannon diversity index across the treatment period. The high-dose rifampicin regimen, HR_35mg/kg_ZE reduced richness only at week 2 (p=0·026), while Faith’s phylogenetic diversity (p=0·0055 at week 2, p=0·015 at week 8, and p=0·014 at week 12) and Shannon diversity index (p=0·027 at week 2, p=0·021 at week 8, and p=0·011 at week 12) were reduced across the follow-up period.

There was no significant reduction of evenness with HR_35mg/kg_ZE across the treatment course. The rifampicin–moxifloxacin regimen, HR_20mg/kg_ZM, had an early effect on all alpha diversity measures, reducing richness (p=0·0007 at week 2 and p=0·0023 at week 8), Faith’s phylogenetic diversity (p=0·0078 at week 2 and p=0·0025 at week 8), Shannon diversity index (p=0·0040 at week 2 and p=0·015 at week 8), and evenness (p=0·015 at week 2). Although there was an early reduction in richness in those given the HR_20mg/kg_ZM regimen, microbiome recovery was observed in all diversity measures at week 12 and from week 8 of treatment for evenness. HR_900mg/kg_ZE only had reduced richness at week 1 (p=0·031) and HR_20mg/kg_ZQ had reduced evenness at week 12 (p=0·0093) of treatment. No significant reduction or increase was observed with HR_10mg/kg_ZQ and HR_1200mg_ZE in all alpha diversity indices across the treatment period (table 2, table 3).

We sought to identify which taxa changed and how they changed under the treatment regimens that induced a significant change in alpha diversity measure. Taxa were sorted based on their relative abundance (abundance), with more than 1% abundance named, while the ones with abundance between 0·0–0·9% were grouped under others. Along the course of treatment, some of the species with more than 1% abundance were reduced to less than 1% and were replaced by those from the others group, whose relative abundance rose to more than 1%. In general, the most abundant species never fell to less than 1% abundance across the treatment period in all regiments (see appendix p 8 for alpha diversity).

Distinctive regimen-induced taxa changes were observed in the microbiota with less than 1% abundance. Considering that the changes in the taxa with more than 1% abundance were less distinctive between regimens, we analysed the taxa with less than 1% abundance (0·01–0·99 relative abundance). In the first-line regimen, HR_600mg_ZE or HR_10mg/kg_ZE, 50 taxa had less than 1% relative abundance before treatment, of which seven (14%) of 50 were reduced to undetectable levels by week 2 of treatment. In the experimental regimens, HR_35mg/kg_ZE and HR_20mg/kg_ZM, there were 56 and 62 pre-treatment taxa at less than 1% relative abundance, of which 24 (45%) of 53 and 32 (52%) of 62 were undetectable by week 2 of treatment, respectively. Gram-negative genera were the most represented in the taxa cleared by regimens, five (71%) of seven for HR_600mg_ZE or HR_10mg/kg_ZE, 12 (50%) of 24 for HR_35mg/kg_ZE, and 19 (59%) of 32 for HR_20mg/kg_ZM, compared with Gram-positive genera, two (27%) of seven in HR_600mg_ZE or HR_10mg/kg_ZE, five (21%) of 24 in HR_35mg/kg_ZE, and six (19%) of 32 in HR_20mg/kg_ZM, cleared by the same regimens (appendix p 9).

**Figure 3:**
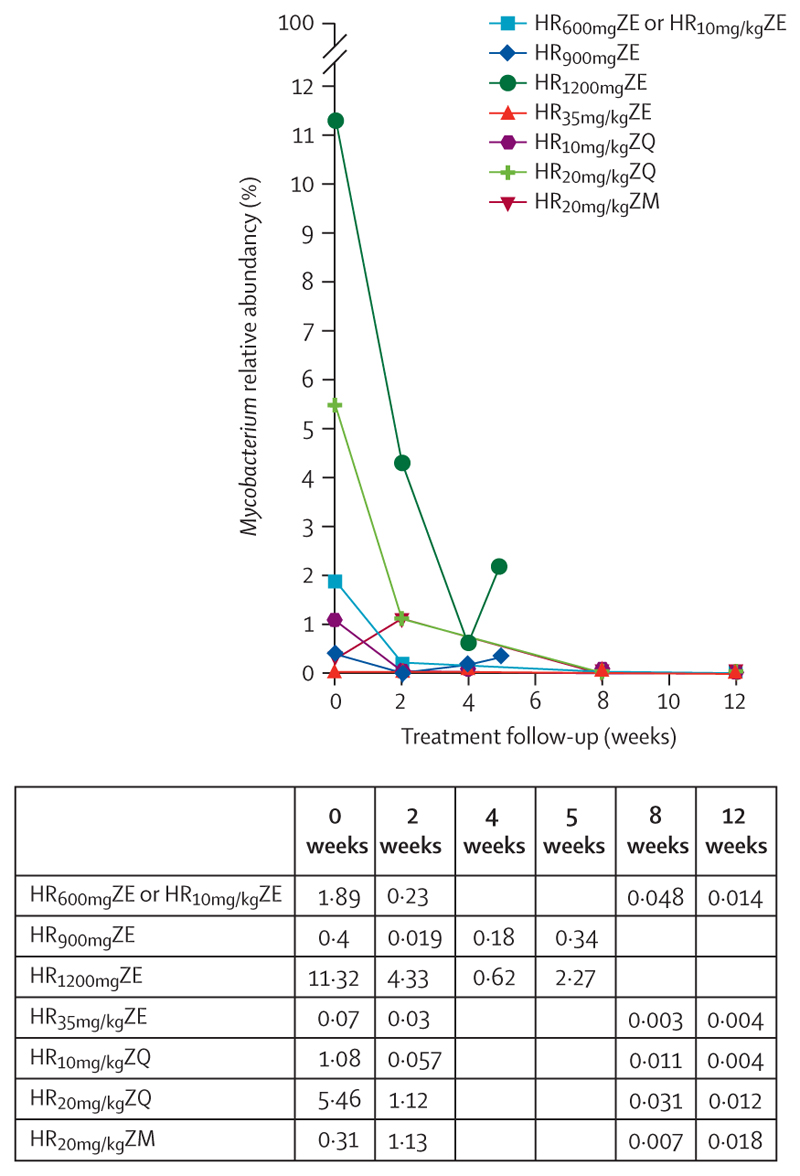
The relative abundance of *Mycobacterium* over the treatment course There was no recovery to pre-treatment levels in all regimens.

The relative abundance of *Mycobacterium* spp continuously reduced and did not recover to pre-treatment levels in all treatment regimens. The reduction in relative abundance of *Mycobacterium* spp was correlated with the *M tuberculosis* bacillary load measured by the ribosomal RNA-based reverse transcriptase quantitative PCR test, TB-MBLA, across the period of treatment (*r*=0·74, 95% CI 0·49–0·87, p<0·0001; figure 3).

**Figure 4:**
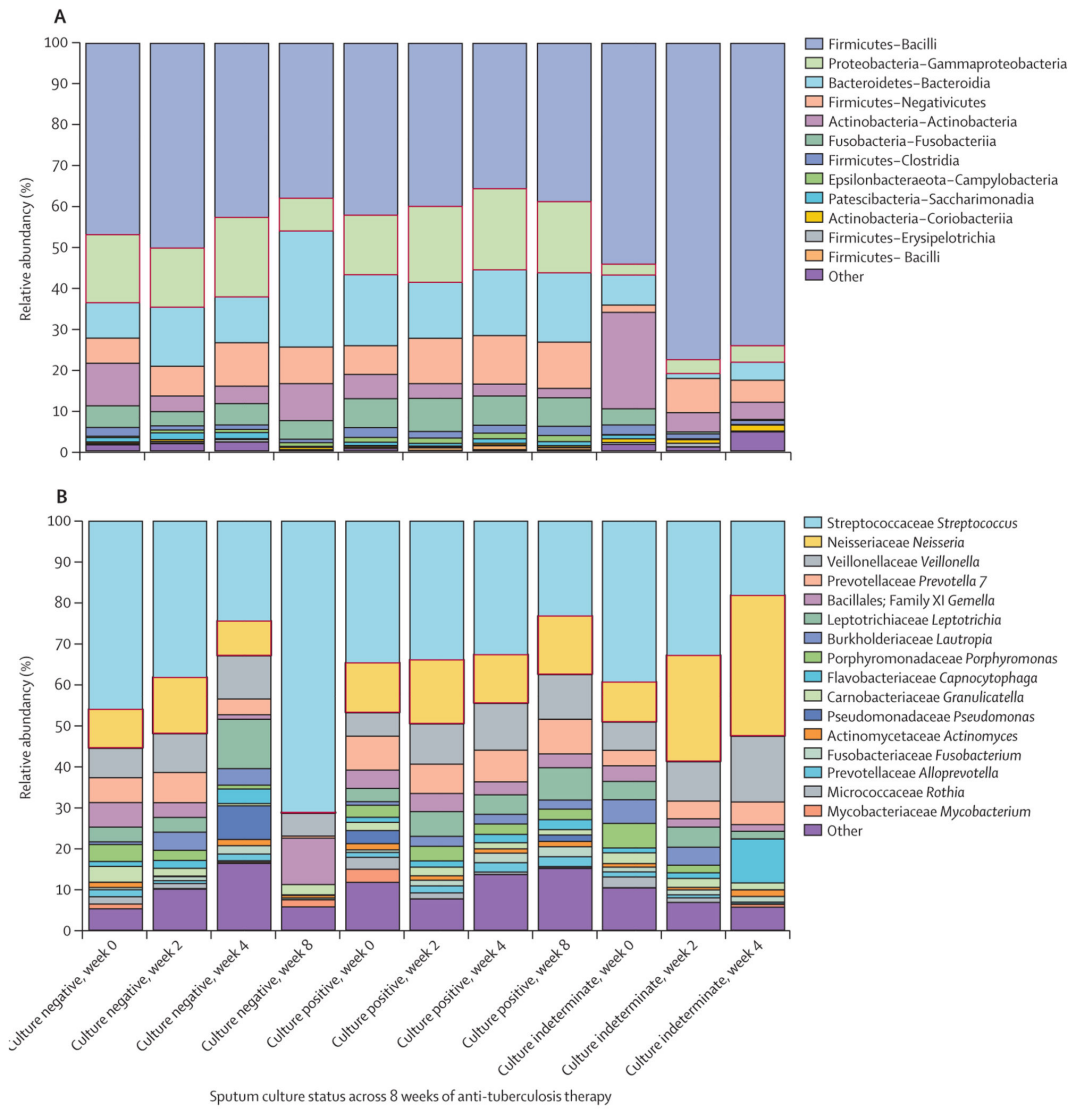
Association of taxa change and treatment outcome at week 8 of treatment (A) Phylum–class level plot showing change in Proteobacteria–Gammaproteobacteria in culture negative, culture positive, and culture indeterminate participants. (B) Genus level association showing changes in genus *Neisseria* among participants in culture negative, culture positive, and culture indeterminate groups. *Neisseria* was reduced by 98% in participants whose culture converted by week 8 of treatment.

Participants were grouped according to their tuberculosis liquid culture results at week 8 of treatment: culture negative (early conversion), culture positive (non-converters), and indeterminate (culture grows a contaminant but tuberculosis cannot be definitively ruled in or out). We observed that the effect of the regimen–microbiome interaction was associated with the culture test outcome (appendix p 10). Contingency plots of their microbiome at phylum–class and family–genus levels revealed Proteobacteria of class Gammaproteobacteria most distinctively changed between the treatment outcome groups. Gammaproteobacteria include the most implicated pathogenic bacteria: *Neisseria* spp, *Pseudomonas* spp, *Moraxella* spp, and *Haemophilus* spp. By week 8 of treatment, abundance of Gammaproteobacteria was substantially reduced, of which genus *Neisseria* was decreased by 98% in sputum samples in the culture negative group (early conversion group). In the non-converted (culture positive) group, Gammaproteobacteria abundance stayed relatively stable, including a 2% increase in genus *Neisseria*. The indeterminate group had a small pre-treatment abundance of Gammaproteobacteria that remained small but was dominated by genus *Neisseria*, which increased by 25% from baseline to week 8 of treatment (figure 4A, B).

## Discussion

In this study, we systematically assessed the effect of different combinations of anti-tuberculosis antibiotics on respiratory microbiomes and the implication of this effect on treatment outcomes. Unlike most studies that use DNA, microbiome was drawn from RNA, enabling us to accurately assess the effect of antibiotics on microbiome viability over the course of treatment. We show that, once exposed to anti-tuberculosis antibiotics, the microbiome diversity and abundance takes a fall and rise pattern akin to that of predator–prey relationships, reflecting the sensitivity and resilience of the microbiome under antibiotic pressure. In a longitudinal follow-up of 12 weeks, a significant reduction in microbiome diversity was observed in the first 2 weeks of treatment, recapitulating the early bactericidal activity of anti-tuberculosis antibiotics. The regimens that contained moxifloxacin and rifampicin 35 mg/kg were responsible for the largest reduction of microbiome diversity that was significantly different from pre-treatment levels. Although most of the taxa recovered, genus *Mycobacterium* did not show recovery, suggesting a unique sensitivity to anti-tuberculosis antibiotics. The trend of *Mycobacterium* spp elimination was measured by TB-MBLA, a reverse transcriptase quantitative qPCR-based assay that specifically quantifies viable bacilli from the *M tuberculosis* complex.^16^ It is important to note that, despite causing tuberculosis disease, *Mycobacterium* was never the most abundant taxa in any of the patients. Future studies could explore the existence of *M tuberculosis* as commensal bacteria, particularly in asymptomatic or healthy individuals, and the threshold or trigger by which *M tuberculosis* becomes pathogenic.

Pre-treatment microbiome was more diverse in the southwest of Tanzania than in north–southeast, suggesting a geographical influence on host microbiome. Geographical location, which encapsulates environment and the kind of diet, has been shown to shape the microbiome acquired.^1^ However, participants in both regions had uneven microbiome, dominated by Firmicutes (*Streptococcus* spp), Bacteroidetes (*Veillonella* spp), Proteobacteria (*Neisseria* spp), and Actinobacteria (*Mycobacterium* spp). Dominance of *Streptococcus* spp, *Viellonela* spp, and *Neisseria* spp in respiratory microbiota have been reported by other studies.^1^

Although all regimens exhibited a reduction in richness, diversity, and evenness, it was significant under HR_20mg/kg_ZM and HR_35mg/kg_ZE regimens, which had an effect across alpha diversity metrics. While the effect of the moxifloxacin regimen was strong until week 8 of treatment, the suppressive effect of HR_35mg/kg_ZE on taxa diversity was sustained up to 12 weeks of treatment, suggesting a long-term impact of rifampicin. These observations are in line with the reported rapid action and sterilising effect of moxifloxacin and rifampicin.^12,17^ Of the two regimens, only the moxifloxacin regimen reduced evenness in the first 2 weeks of treatment. The HR_10mg/kg_ZQ regimen also had an effect on phylogenetic diversity and evenness in the same 2-week period. By contrast, there was an increase in evenness by taxa under the standard HR_600mg_ZE or HR_10mg/kg_ZE from week 2 through to week 12 of treatment. The insignificant reduction of evenness by HR_35mg/kg_ZE and gain exhibited by HR_600mg_ZE or HR_10mg/kg_ZE are an indication that the rifampicin bactericidal effect might be evenly spread across taxa, which consequently evens out taxa distribution rather than elimination. The effect might be different at a rifampicin dose higher than 35 mg/kg. The PanACEA MAMS-TB clinical trials found the HR_35mg/kg_ZE regimen more effective at causing stable culture conversion and recommended it for phase 3 studies. Since HIGHRIF study 2 found that a fixed dose of rifampicin 1200 mg was not superior to standard rifampicin 600 mg at causing culture conversion, it is plausible to speculate that the effect we have observed in the HR_20mg/kg_ZE regimen is most likely contributed by moxifloxacin.

A few taxa (n=12) had relative abundance of more than 1%, leaving the majority with an abundance of less than 1% (0·10–0·99%). The over 1% abundant taxa remained relatively stable, with minimal reduction by antibiotic action. There was a notable reduction in relative abundance under the moxifloxacin regimen, where *Streptococcus* spp was reduced by 15% and displaced by *Prevotella* as the second most abundant and *Neisseria* was reduced by 14% reduction from second to seventh position, taking up to week 12 to recover its abundance. By contrast, there was a 7% increase in *Streptococcus* spp and a 4% increase in *Neisseria* spp during the rifampicin 35 mg/kg regimen in the first 2 weeks of treatment and only 3% and 1% reduction of the same genera during the HR_600mg_ZE or HR_10mg/kg_ZE regimens. This effect demonstrates the strong early bactericidal activity of the moxifloxacin containing regimen. The strong early bactericidal activity implies that supplementing rifampicin with moxifloxacin has stronger and faster antibacterial action than just increasing rifampicin dose alone.

The relatively stable taxa that had more than 1% abundance could not explain the reduction in richness and diversity observed in the first 2 weeks of treatment. A deeper analysis of the taxa that had a relative abundance of less than 1% abundance revealed the members of the community had reduced in abundance to below detection by week 2 of treatment. More than half of the taxa that were reduced to sub-detectable levels were Gram-negative bacteria (36 [57%] of 63, of which 19 [53%] were reduced under the regimen containing moxifloxacin). It has long been demonstrated that fluoroquinolones, such as moxifloxacin, have strong action against Gram-negative bacteria (including *Neisseria meningitidis*), Gram-positive *Streptococcus pnuemoniae*, and generally anaerobic bacteria.^18–20^

Although the rest of the taxa recovered during treatment, members of the genus *Mycobacterium* did not recover across all regimens, suggesting some form of selective elimination. A similar reduction of *Mycobacterium* spp with regard to recovery of other taxa was reported by Katete and colleagues^21^ in patients with tuberculosis given the standard HR_10mg/kg_ZE regimen. The sustained elimination of *Mycobacterium* spp might be explained by constituents of the anti-tuberculosis regimen (isoniazid, pyrazinamide, and ethambutol), which act specifically on *Mycobacterium* spp possibly enhanced by broad-spectrum rifampicin or moxifloxacin.^22,23^

We explored whether antibiotic–microbiome interactions impacted tuberculosis treatment outcomes. Proteobacteria genus *Neisseria* was reduced to a sub-detectable level in patients who converted to negative tuberculosis culture by week 8 of treatment. By contrast, there was a 2% rise in *Neisseria* spp in patients who remained culture positive and a 25% rise in *Neisseria* spp in patients who remained indeterminate by week 8 of treatment.

The sample size is small when divided by the number of analysed regimens. However, the pre-treatment core microbiome covered in this study is consistent with and representative of the respiratory microbiota composition published by other studies conducted in east Africa and outside Africa.^24,25^ This implies that the core microbiome is similar between participants and might not vary much irrespective of the number of participants. Secondly, our study did not examine from which source the recovering taxa came from. Additional studies should investigate whether recovery was due to regrowth of suppressed taxa or due to replenishment from dietary sources and investigate the impact on long-term clinical and health outcomes.

In summary, we have shown that different anti-tuberculosis regimens and dosages have different effects on the sputum microbiome. The standard first-line regimen, HR_600mg_ZE or HR_10mg/kg_ZE, appeared to affect the microbiome only marginally, causing an increase in taxa evenness and no significant reduction of diversity. Within the backbone of the standard regimen, increasing the dose of rifampicin alone to 35 mg/kg achieved a significant reduction of the microbiome, which did not recover to pre-treatment level by month 3 of treatment follow-up. A lower rifampicin dose of 20 mg/kg supplemented with 400 mg moxifloxacin resulted in a significant reduction of microbiome diversity, which recovered to pre-treatment level by month 3 of treatment. The drug, SQ109, appeared to have added no significant value to the performance of anti-tuberculosis regimen or its effect on the microbiome. Most importantly, *M tuberculosis* did not show recovery across regimens, an effect that implies that novel optimal anti-tuberculosis regimens could shorten treatment course without irreversible damage to the beneficial respiratory microbiome. Furthermore, large-scale longitudinal studies will be needed to ascertain whether it is only *M tuberculosis* that is eliminated and what implications this has on the recovering microbiome and treatment outcome.

## Supplementary Material

Supplementary Material

## Figures and Tables

**Table 1:** Drug dosing and duration under the PanACEA MAMS-TB and HIGHRIF study 2 clinical trials

	Dosage and duration
**PanACEA MAMS-TB clinical trial^12^**	
Group 1 (HR_35mg/kg_ZE)	35 mg/kg per day rifampicin, 15–20 mg/kg per day ethambutol, 5 mg/kg per day isoniazid, and 25 mg/kg per day pyrazinamide for 12 weeks followed by 5 mg/kg per day isoniazid and 10 mg/kg per day rifampicin for 14 weeks
Group 2 (HR_20mg/kg_ZM)	20 mg/kg per day rifampicin, 400 mg/kg per day moxifloxacin, 5 mg/kg per day isoniazid, and 25 mg/kg per day pyrazinamide for 12 weeks followed by 5 mg/kg per day isoniazid and 10 mg/kg per day rifampicin for 14 weeks
Group 3 (HR_20mg/kg_ZQ)	20 mg/kg per day rifampicin, 300 mg/kg per day SQ109, 5 mg/kg per day isoniazid, and 25 mg/kg per day pyrazinamide for 12 weeks followed by 5 mg/kg per day isoniazid and 10 mg/kg per day rifampicin for 14 weeks
Group 4 (HR_10mg/kg_ZQ)	10 mg/kg per day rifampicin with 300 mg SQ109, 5mg/kg per day isoniazid, and 25 mg/kg per day pyrazinamide for 12 weeks followed by 5 mg/kg per day isoniazid and 10 mg/kg per day rifampicin for 14 weeks
Control (HR_10mg/kg_ZE)	10 mg/kg rifampicin, 5 mg/kg isoniazid, 25 mg/kg pyrazinamide, and 15–20 mg/kg ethambutol daily
**HIGHRIF study 2 clinical trial^13^**	
600 mg (HR_600mg_ZE)	Four capsules of fixed-dose combination dose (each capsule containing rifampicin 150 mg, isoniazid 75 mg, pyrazinamide 400 mg, and ethambutol 275 mg) plus two capsules of placebo
900 mg (HR_900mg_ZE)	Four capsules of fixed-dose combination dose (each capsule containing rifampicin 150 mg, isoniazid 75 mg, pyrazinamide 400 mg, and ethambutol 275 mg) plus one capsule with 300 mg rifampicin plus one capsule of placebo
1200 mg (HR_1200mg_ZE)	Four capsules of fixed-dose combination (each capsule containing rifampicin 150 mg, isoniazid 75 mg, pyrazinamide 400 mg, and ethambutol 275 mg) plus two capsules with 300 mg rifampicin
All participants	Each participant received 300 mg isoniazid and 600 mg rifampicin daily for 4 months of post-intensive phase treatment

**Table 2:** Mann-Whitney test of the difference between pre-treatment and post-treatment microbiome under different regimens at weeks 2, 8, and 12

	Observed ASVs				Faith’s phylogenetic diversity				Shannon diversity index				Evenness		
	Week 2	Week 8	Week 12		Week 2	Week 8	Week 12		Week 2	Week 8	Week 12		Week 2	Week 8	Week 12
**HR_600mg_ZE or HR_10mg/kg_ZE**															
Baseline (n=19)	88	88	88		8·02	8·02	8·02		4·68	4·68	4·68		0·72	0·72	0·72
Follow-up (n=16–26)	66	88	71·5		7·04	7·94	7·16		4·38	4·97	4·83		0·76	0·76	0·77
p value	0·090	0·92	0·61		0·09	>0·99	0·50		0·97	0·27	0·50		0·035*	0·024*	0·034*
**HR_35mg/kg_ZE**															
Baseline (n=9)	102	102	102		9·23	9·23	9·23		5·49	5·49	5·49		0·81	0·81	0·81
Follow-up (n=8–9)	63	74·5	72		6·58	7·66	7·6		4·76	4·83	4·78		0·8	0·75	0·76
p value	0·026*	0·070	0·089		0·0055*	0·015*	0·014*		0·027*	0·021*	0·011*		0·54	0·17	0·063
**HR_10mg/kg_ZQ**															
Baseline (n=7)	80	80	80		7·55	7·55	7·55		4·75	4·75	4·75		0·74	0·74	0·74
Follow-up (n=8)	77·5	61	58·5		6·88	6·33	6·75		4·79	4·16	4·37		0·75	0·71	0·74
p value	0·63	0·29	0·52		0·34	0·39	0·61		0·96	0·78	0·87		0·39	0·78	0·55
**HR_20mg/kg_ZQ**															
Baseline (n=8)	98	98	98		8·96	8·96	8·96		4·96	4·96	4·96		0·75	0·75	0·75
Follow-up (n=7–9)	65	80	76		6·92	7·39	7·5		3·85	4·22	4·61		0·64	0·69	0·71
p value	0·16	0·15	0·34		0·094	0·075	0·19		0·014*	0·059	0·24		0·0093*	0·14	0·33
**HR_20mg/kg_ZM**															
Baseline (n=9)	82	82	82		7·96	7·96	7·96		4·77	4·77	4·77		0·75	0·75	0·75
Follow-up (n=7–9)	31	38	50		3·76	5·08	6·032		2·42	3·51	4·41		0·55	0·71	0·73
p value	0·0007*	0·0023*	0·29		0·0078*	0·0025*	0·41		0·0040*	0·015*	0·41		0·015*	0·24	0·76

Baseline and follow-up are in median, unless specified. Except for upward trend of evenness under the HR_600mg_ZE or HR_10mg/kg_ZE regimen, in most cases microbiome took a downward trend that was significant in the HR_35mg_ZE or HR_20mg/kg_ZM regimens. See table 1 for regimen details. ASV=amplicon sequence variants.

* p<0·05.

**Table 3:** Mann-Whitney test of the difference between pre-treatment and post-treatment microbiome under different regimens at weeks 1, 4, and 5

	Observed ASVs				Faith’s phylogenetic diversity				Shannon diversity index				Evenness		
	Week 1	Week 4	Week 5		Week 1	Week 4	Week 5		Week 1	Week 4	Week 5		Week 1	Week 4	Week 5
**HR_900mg_ZE**															
Baseline (n=6)	64·5	64·5	64·5		6·52	6·52	6·52		3·84	3·84	3·84		0·67	0·67	0·67
Follow-up (n=7–14)	26·S	32	41		3·66	3·88	4·58		3·02	3·39	3·79		0·65	0·67	0·71
p value	0·031*	0·25	0·33		0·091	0·18	0·35		0·091	0·23	0·85		0·90	>0·99	0·28
**HR_1200mg_ZE**															
Baseline (n=7)	30	30	30		3·67	3·67	3·67		3·48	3·48	3·48		0·74	0·74	0·74
Follow-up (n=6–11)	23	27	46·5		3·13	3·58	5·19		3·12	3·01	4·01		0·64	0·66	0·72
p value	0·20	0·29	0·63		0·29	0·37	0·95		0·15	0·21	0·95		0·33	0·25	0·73

Baseline and follow-up are in median, unless specified. Except for upward trend of evenness under the HR_600mg_ZE or HR_10mg/kg_ZE regimen, in most cases microbiome took a downward trend that was significant in the HR_35mg_ZE or HR_20mg/kg_ZM regimens. See table 1 for regimen details. ASV=amplicon sequence variants.

* p<0·05.

## Data Availability

Sequence data were deposited and are publicly available in the US National Center for Biotechnology Information (NCBI) Sequence Read Archive (SRA) under the BioProject (PRJNA729425). Excel spreadsheets of Alpha diversity data, analysis, and data dictionary are hosted at the University of St Andrews and will be retained for 10 years as per ethical approval. Data can be accessed by contacting the corresponding authors and meeting the ethical requirements by which the data were collected.

## Research in context

**Evidence before this study**

Tuberculosis is treated with combinations of antibiotics in first-line and second-line regimens, depending on the drug sensitivity profile of the tuberculosis bacteria. We asked what impact these antibiotics, particularly the new clinical trial regimens, have on the respiratory microbiome and treatment outcome. We assessed high-dose rifampicin plus novel combinations, including moxifloxacin and SQ109, for efficacy in treating tuberculosis in two clinical trials, HIGHRIF study 2 and PanACEA MAMS-TB clinical trials. These studies showed that high-dose rifampicin was safe and efficacious and that including moxifloxacin increased early mycobactericidal activity without affecting safety. We searched PubMed, MEDLINE, and EMBASE using search terms “respiratory microbiome”, “microbiota”, “anti-tuberculosis antibiotics”, “tuberculosis treatment response”, and “antibiotic impact on microbiome” for studies published between July 1, 2003, and March 30, 2022, in the English language. The available literature described the microbiome of people who were healthy compared with people with tuberculosis and the effects of the first-line anti-tuberculosis regimen on the respiratory microbiota. No information was available on the new combinations of antibiotics to treat tuberculosis. Therefore, we assessed the impact of the new regimens on the sputum microbiome in comparison with the standard first-line regimen.

**Added value of this study**

This study has revealed the value and knowledge of using RNA, rather than DNA, in studying microbiome and how it responds to antibiotic therapy. RNA is a molecule that reflects cell viability, and, thus, is crucial for investigating the effect of antibiotics on microbiota. These findings have improved understanding of how new anti-tuberculosis regimens affect the microbiome, particularly the high sensitivity exhibited by the microbiome in the first two weeks of treatment.

**Implications of all the available evidence**

Although the microbiome recovers following exposure to antibiotics, it happens at different rates under different regimens. Broad-spectrum antibiotics in regimens have a large depressing effect on the microbial community, but this can be reduced by optimising the dosage. Therefore, it is crucial that microbiome analysis is considered as one of the parameters to evaluate and approve new antibiotics or treatment combinations. Future studies considering large sample size and long-term clinical outcomes will be crucial in establishing microbiome analysis as a drug safety parameter to include in clinical trials. Further investigation is needed to ascertain whether recovery of the microbiome while still under antibiotic pressure is influenced by replenishment from dietary sources or acquisition of genes for antibiotic resistance.
