# Supplementary material for: Effect of seven anti-tuberculosis treatment regimens on sputum microbiome: a retrospective analysis of the HIGHRIF study 2 and PanACEA MAMS-TB clinical trials
Source: Lancet Microbe. Author manuscript; Available in PMC 2025 Feb 16. (PMC7617392; doi:10.1016/S2666-5247(23)00191-X)
Supplement: Supplementary Material [file EMS202205-supplement-Supplementary_Material.pdf]

THE LANCET
Microbe

Supplementary appendix

This appendix formed part of the original submission and has been peer reviewed. We post it as supplied by the authors.

Supplement to: Musisi E, Wyness A, Eldirdiri S, et al. Effect of seven anti-tuberculosis treatment regimens on sputum microbiome: a retrospective analysis of the HIGHRIF study 2 and PanACEA MAMS-TB clinical trials. *Lancet Microbe* 2023; published online Oct 10. https://doi.org/10.1016/S2666-5247(23)00191-X.

Supplementary material

Effect of seven anti-tuberculosis treatment regimens on sputum microbiome: a retrospective analysis of the High-Rifampicin II- and Multi-Arm-Multi-Stage clinical trials

Emmanuel Musisi MSc[1], Adam Wyness PhD[1,2], Sahar Eldirdiri MD[3], Evelin Dombay PhD[1], Bariki Mtafya PhD[1,4], Nyanda E. Ntinginya PhD[4], Norbert Heinrich PhD[5], Gibson S. Kibiki PhD[6,7], Michael Hoelscher PhD[5,8], Martin Boeree PhD[9], Rob Aarnoutse PhD[10], Stephen H. Gillespie DSc[1] and Wilber Sabiiti PhD[1] on behalf of the PanACEA consortium

Supplementary text 1: Clean-up of the Amplicons

Complementary DNA Amplicons were homogenised with 56 µl of the magnetic beads and incubated at room temperature for 5 min during which cDNA got bound to the magnetic beads· The cDNA amplicon-beads complex was separated from other mixture content by placing it in the magnetic plate for 2 min· The resulting clear supernatant was pipetted off and discarded leaving the cDNA amplicon-beads complex behind· This complex was washed twice using 80% ethanol· While on the magnetic stand, the washed beads were air dried for 10 min· Amplicons were eluted from the beads buy incubating with 27·5µl of 10mM Tris HCL; pH 8·5· While on the magnetic stand, the supernatant which contained clean cDNA amplicons were transferred to another clean PCR plate·[3] (Supplementary figure S1)

Supplementary figure S1: 16S rRNA V3/V4 gene library of the sputum microbiome

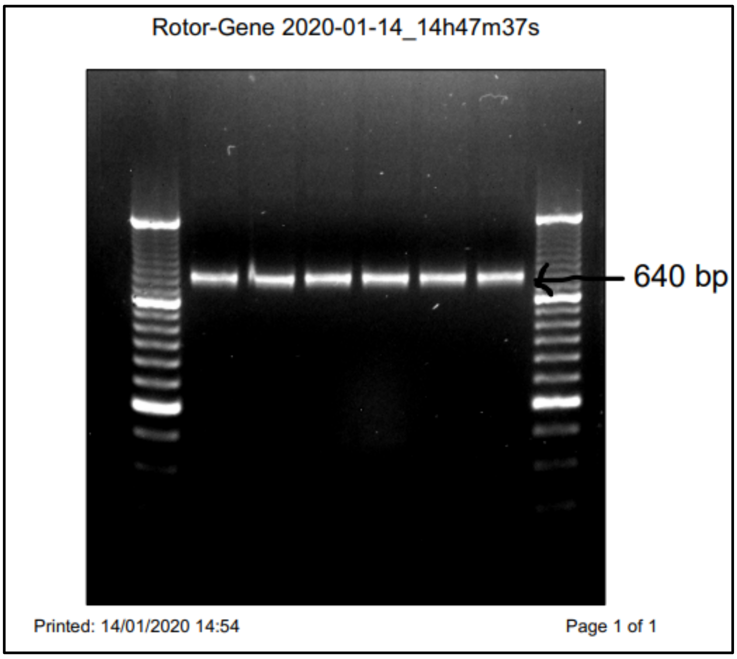

16S rRNA V3/V4 gene library of the sputum microbiome. Agarose gel concentration was 2%· Electrophoreses conditions were: 97 Voltage, 400Amp for 60 min· SYBRsafe DNA dye was used and 50 bp ladder (Thermo Fisher Scientific, UK) was used as a reference·

Supplementary text 2: Demographics of study participants

A total of 397 pre- and post-treatment sputum samples from 65 bacteriologically confirmed pulmonary TB patients were analysed over a 12-week treatment period. Sixteen (24%) and 49 (76 %) of the patients were from northeast- and southwest- Tanzania, respectively. Of the 49 southeast Tanzania participants, 69% were male and the overall median age was 34 (range 19 – 65 years). No disaggregated gender and age data was available for the north-southeast Tanzania participants, however, Aarnouste et al., reported median age of 33·5 and 90% male of the 150 participants of the study from which the samples were obtained.

Pre-treatment microbiome by sample origin at phylum-class level (Supplementary figure S2)

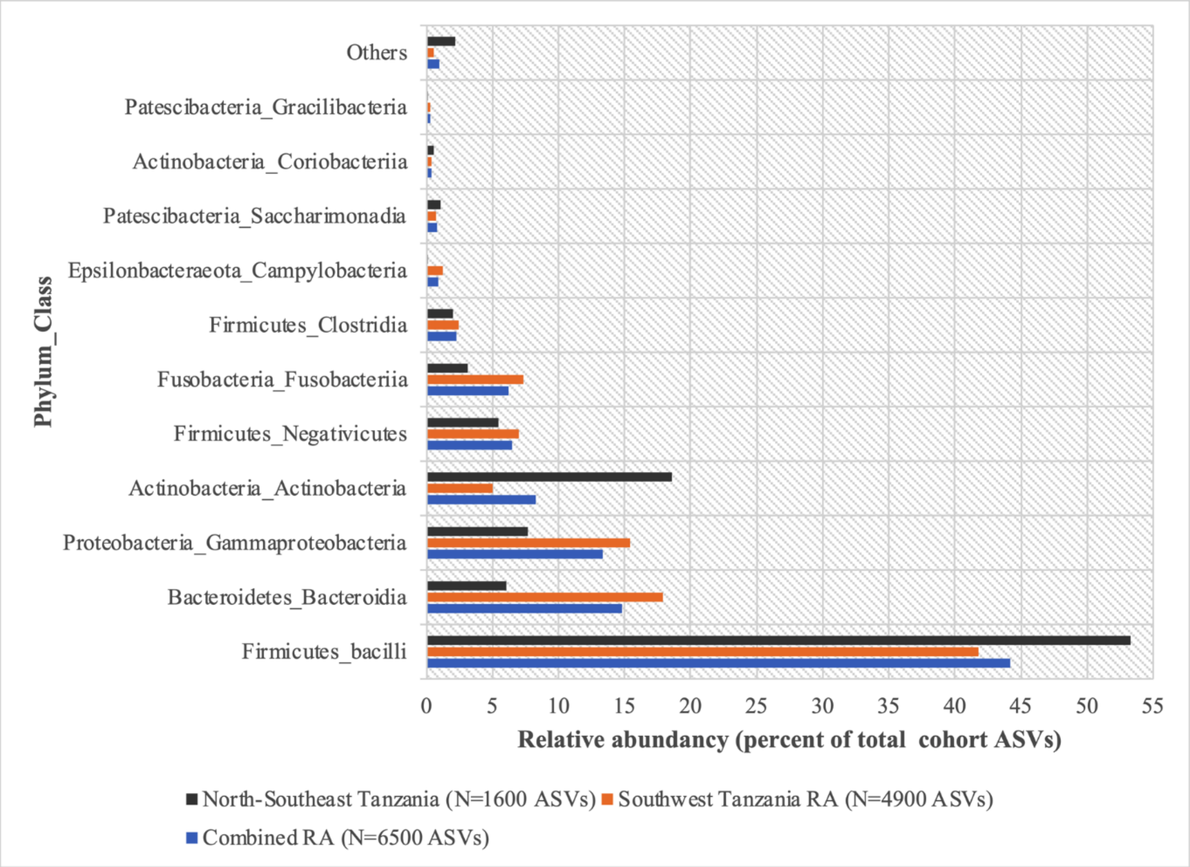

Supplementary Figure S2: Pre-treatment microbiome diversity at phylum and class level divided by the region of origin. North-southeast Tanzania (black bars), Southwest Tanzania (orange bars) and combination of the 2 regions (blue bars). There was an over-representation of Firmicutes and Actinobacteria in North-southeast region.

Pre-treatment microbiome by sample origin at genus level (Supplementary figure S3).

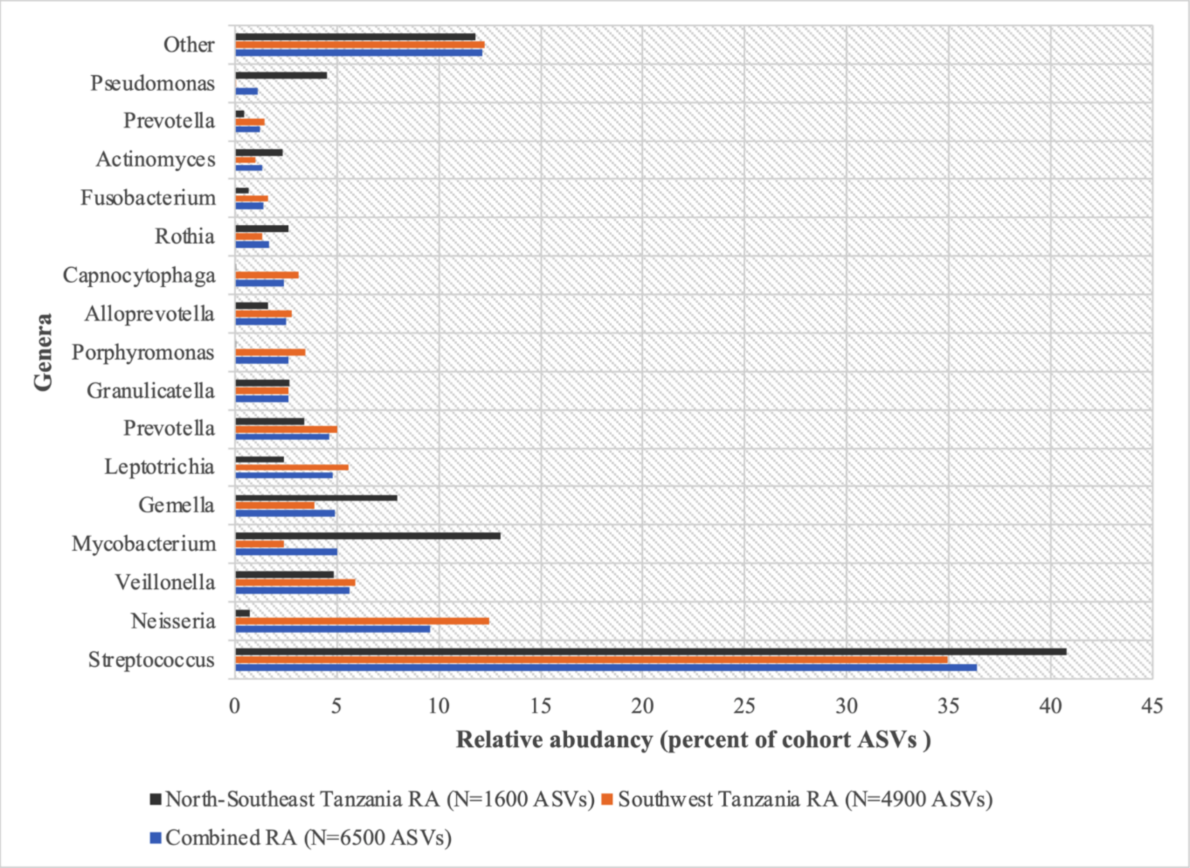

Supplementary figure S3: Pre-treatment microbiome diversity at genus level divided by region of origin. North-southeast Tanzania (black bars), Southwest Tanzania (orange bars) and combination of the 2 regions (blue bars). There was an over representation of genus *Mycobacterium* in North-southeast region.

Impact of selected regimens on taxa with over 1% relative abundance

Under standard regimen HRZE, there were 12 genera with ≥1% abundancy in the pre-treatment microbiome of which *Streptococcus,* 47% was the most abundant, followed by the other group, 16%, *Neisseria* 10% and *Gemella* 6%. At week 2 of treatment, the first three retained their positions but with a drop in RA to 43%, 10%, 9·4% whilst *Gemella* fell to 3·8% and was replaced by *Veillonella*, 9·1% in the 4th position. *Streptococcus* dropped to 34%, others group rose to 20%, *Veillonella* rose to 13% taking over *Neisseria*, and *Prevotella* became the 4th abundant genus, 8% by week 8 of treatment. By week 12 of treatment, *Streptococcus* dropped to 33%, *Neisseria* bounced back to 20% taking over others group, 12% and *Veillonella* at 11%. Although the 'other groups' changed from 16% at baseline to 10%, 20% and 12% by week 2, 8 and 12 respectively, they retained 2nd most abundant position except week 12 where they

dropped to 3rd position. *Mycobacterium*, 2% abundancy at baseline dropped below one percent and never recovered till end week 12 (Supplementary figure S4).

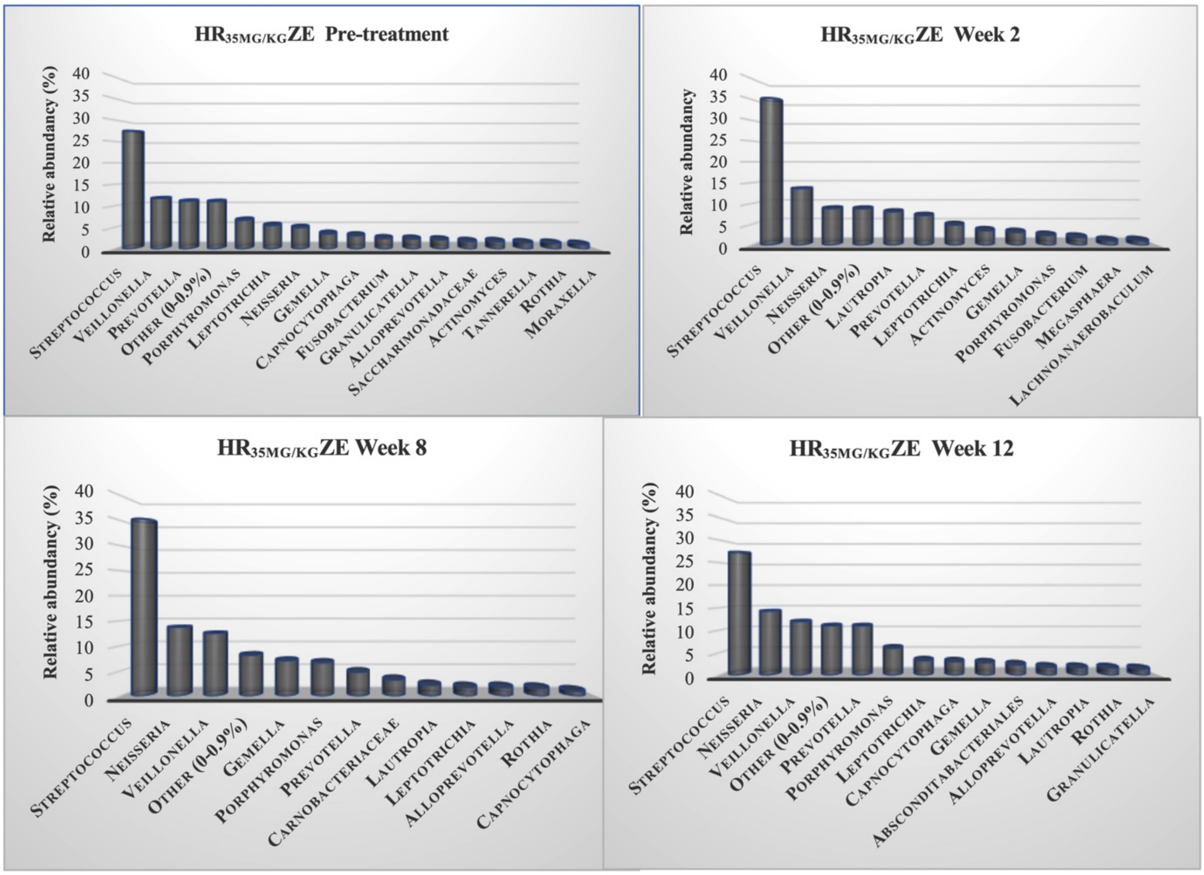

Supplementary figure S4: The taxa and how they changed under treatment with first-line regimen $HR_{600mgor10mg/kg}ZE$. The 'others' group retained 2nd position of abundancy until week 8 of treatment.

In the highest-dose rifampicin regimen, HR35ZE, pre-treatment microbiome was comprised of 16 named genera that had ≥1% abundancy. The most abundant pre-treatment genera were *Streptococcus* 28%, *Veillonella* 12%, and *Prevotella* 11·4% plus the 'others' group at 11·1%. By week 2 of treatment, the genera with ≥1% abundancy had dropped to 11 of which *Streptococcus* 35% and *Veillonella* 14% increase in abundancy while *Prevotella* was replaced by *Neisseria* 9%. Although the 'Others' group retained the 4th position throughout treatment with this regimen, its abundancy changed from 11% to 9%, 8% and 11% by week 2, 8 and 12 of treatment. *Porphyromonas* 7% in the 5th position dropped to 10th position with 2% abundancy by week 2 of treatment but recovered to 6th position with 7% and 6% abundancy by week 8 and 12 of treatment (Supplementary figure S5).

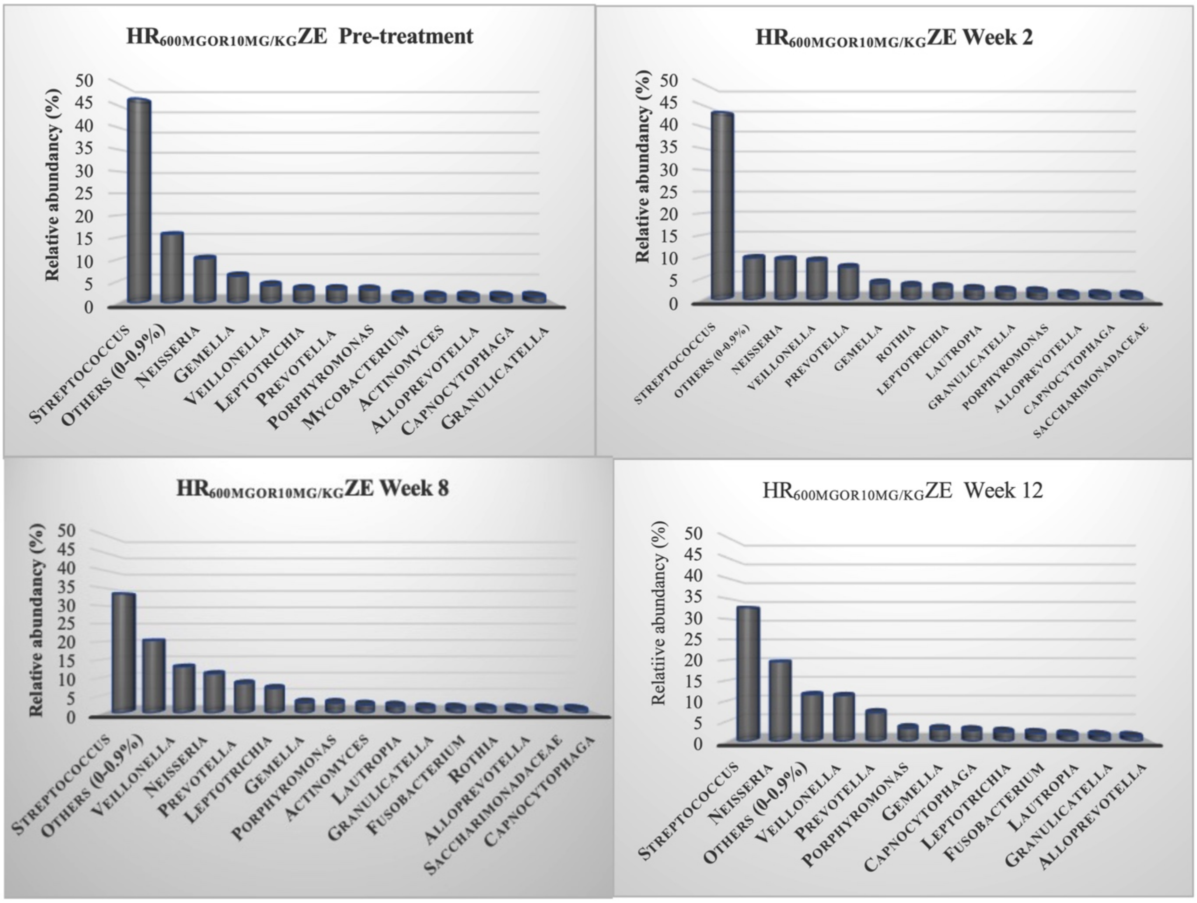

Supplementary Figure S5: The taxa and how they changed under treatment with high dose rifampicin regimen $HR_{35mg/kg}ZE$. The 'others' group retained 4th position of abundancy until week 12 of treatment whilst Porphyromonas was significantly reduced in the first 2 weeks of treatment.

For the high-dose rifampicin-moxifloxacin regimen, the ≥1% pre-treatment microbiome was comprised of 11 genera plus the 'others' group. The most abundant was *Streptococcus*, 40% followed by *Neisseria*, 21%, the others group, 13% and *Rothia* at 5%. By week 2 of treatment, the total number of genera with ≥1% abundancy reduced to eight. Streptococcus dropped to 25% RA and was replaced by *Prevotella*, 27% abundancy in the first position. *Neisseria* was replaced by *Streptococcus*, in the 2nd most abundant position while *Gemella* 13% abundancy and *Veillonela* 10% replaced the 'others' group and *Rothia* in the 3rd and 4th position, respectively. The 'others' group dropped 7% abundancy to 6th position while *Rothia* dropped to below 1% RA and only recovered to 2% abundancy by week 12 of treatment. *Streptococcus* recovered to 35%- and 33%- RA by week 8 and 12 respectively but not to pre-treatment level. In contrast, *Neisseria* dropped to 7% and 4% RA by week 2 and 8 respectively but recovered to 21% RA consistent with pre-treatment level by week 12 of treatment. *Mycobacterium* that was detectable at 1·13% RA at week 2 of treatment, dropped to <1% and never recovered until of end of treatment follow-up (Supplementary figure S6).

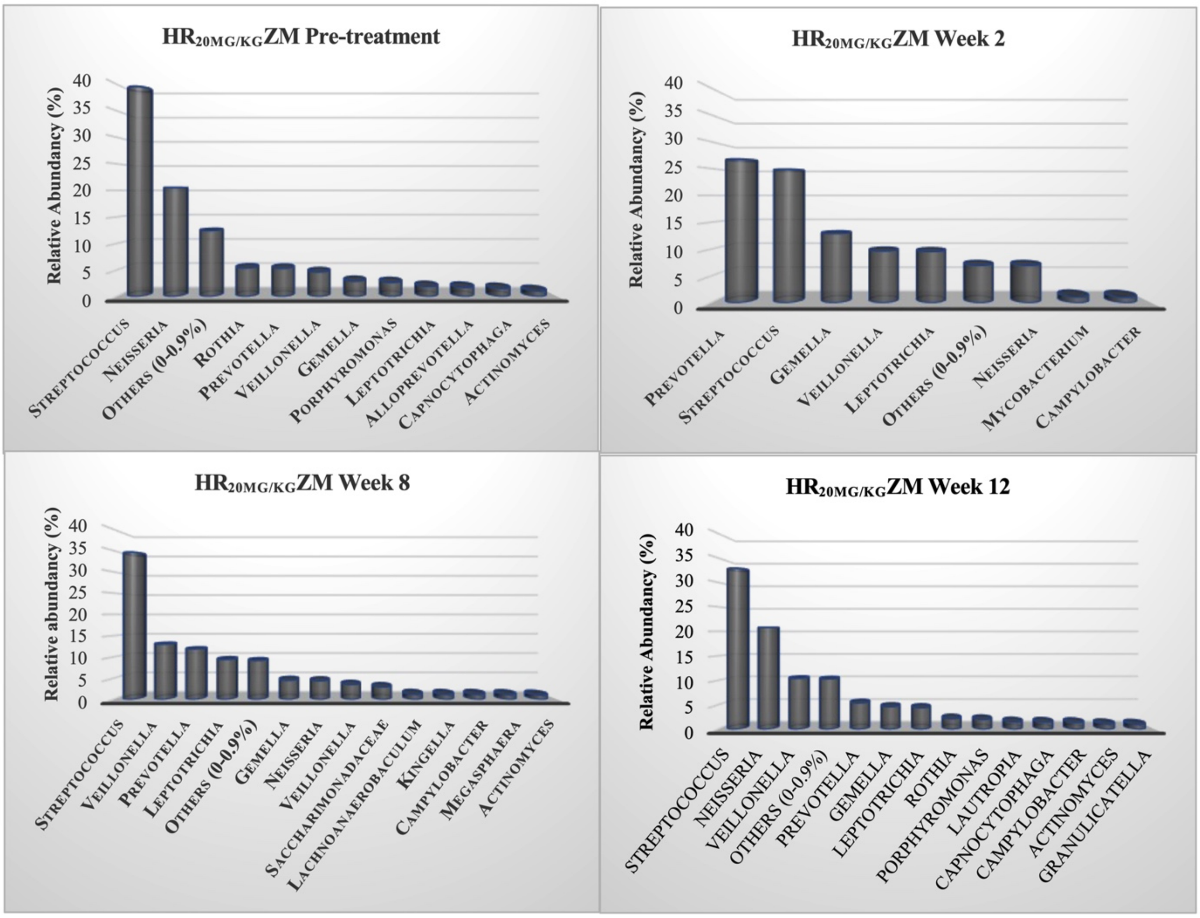

Supplementary figure S6: The taxa and how they changed under treatment with high dose rifampicin-moxifloxacin regimen $HR_{20mg/kg}ZM$. The others group was highly suppressed in the 1st two weeks of treatment. Unlike other regimens, *Streptococcus* abundancy was significantly reduced and never recovered to pre-treatment level over the treatment follow-up period.

Numerical representation of figures S4-S6 above (Supplementary Table 1)

	Relative abundance of taxa (%) over treatment period											
**Regimen**	**$HR_{600mg or 10mg/kg}ZE$**				**$HR_{35mg/kg}ZE$**				**$HR_{20mg/kg}ZM$**			
**Treatment follow-up**	**Wk 0**	**Wk 2**	**Wk 8**	**Wk 12**	**Wk 0**	**Wk 2**	**Wk 8**	**Wk 12**	**Wk 0**	**Wk 2**	**Wk 8**	**Wk 12**
Streptococcus	46.6	43.4	33.52	33.00	27.49	35.33	35.35	27.44	39.57	24.86	34.61	32.90
Others (0-0.9%)	15.73	9.7	20.38	11.51	11.10	8.80	8.17	11.03	12.45	7.01	9.21	10.32
Neisseria	10.14	9.4	11.03	19.61	4.99	8.82	13.74	14.26	20.54	7.00	4.43	20.81
Gemella	6.16	3.8	2.91	2.99	3.55	3.13	7.07	2.82	2.94	12.93	4.56	4.65
Veillonella	4.03	9.1	12.98	11.28	11.78	13.63	12.53	11.96	4.67	9.68	16.61	10.43
Leptotrichia	3.13	2.9	6.95	2.30	5.49	4.89	1.78	3.28	1.92	9.57	9.44	4.44
Prevotella	3.11	7.5	8.32	7.09	11.16	7.17	4.80	11.02	5.35	26.76	11.90	5.41
Porphyromonas	3.04	1.9	2.80	3.22	6.73	2.42	6.69	6.05	2.71	*	*	2.05
Mycobacterium	1.77	*	*	*	-	-	-	-	-	1.13	*	*
Actinomyces	1.64	*	2.29	*	1.78	3.55	*	*	1.11	*	1.12	1.19
Alloprevotella	1.59	1.2	1.26	1.21	2.17	*	1.78	1.83	1.77	*	*	*
Capnocytophaga	1.54	1.2	1.08	2.71	3.14	*	1.03	3.05	1.56	*	*	1.50
Granulicatella	1.52	2.1	1.53	1.47	2.35	*	*	1.46	-	-	-	1.10
Fusobacterium	-	-	1.43	1.99	2.47	1.95	*	*	-	-	-	-
Lautropia	-	2.4	2.01	1.64	-	8.04	2.20	1.75	-	-	-	1.56
Rothia	-	3.2	1.35	*	1.37	*	1.60	1.68	5.41	*	*	2.21
Saccharimonadaceae	-	1.1	1.17	*	1.80	*	*	*	-	-	3.00	*
Absconditabacteriales	-	-	-	-	-	-	-	2.36	-	-	-	-
Carnobacteriaceae	-	-	-	-	-	-	3.26	*	-	-	-	-
Lachnoanaerobaculum	-	-	-	-	-	1.13	*	*	-	-	-	-
Megasphaera	-	-	-	-	-	1.15	*	*	-	-	1.25	*
Moraxella	-	-	-	-	1.12	*	*	*	-	-	1.30	*
Tannerella	-	-	-	-	1.50	*	*	*	-	-	-	-
Campylobacter	-	-	-	-	-	-	-	-	-	1.06	1.29	1.43
Kingella	-	-	-	-	-	-	-	-	-	-	1.29	*

Supplementary table 1: Alpha diversity changes of taxa with over 1% relative abundance under control regimen HRZE and HR35ZE and HR20ZM described in supplementary figures S4 to S6 in the main text· *Taxa with over 1% RA in previous sampling visit but were reduced to below 1%. -Taxa that had below 1% RA at baseline (pre-treatment stage).

The impact of regimens on low abundant microbes (less than 1% relative abundancy.

HRZE Taxa<1% RA at baseline = 50			HR35ZE Taxa <1 RA% at baseline = 53			HR20ZM Taxa <1% RA at baseline = 62		
**Detected at baseline but not at week 2**	**Gram stain**	**Detected at week 2 but not at baseline**	**Detected at baseline but not at week 2**	**Gram stain**	**Detected at week 2 but not at baseline**	**Detected at baseline but not at week 2**	**Gram stain**	**Detected at week 2 but not at baseline**
						Absconditabacteriales (SR1)	ND	Bifidobacteriaceae
Bacillus	Positive	*Butyrivibrio*	*Actinobacillus*	Negative	*Alloprevotella*	*Actinobacillus*	Negative	*Bifidobacterium*
Enterobacter	Negative	*Corynebacterium*	*Aggregatibacter*	Negative	*Granulicatella*			
Halioglobus	Negative	Defluviitaleaceae UCG-011	*Bergeyella*	Negative	*Tannerella*	*Actinobacillus porcinus*	Negative	*Bradyrhizobium*
Klebsiella	Negative	*Dialister*	*Butyrivibrio*	Negative		*Bergeyella*	Negative	*Capnocytophaga*
Lactococcus	Positive	*Filifactor*	*Candidatus Saccharimonas*	N/D		*Cardiobacterium*	Negative	*Enterococcus*
Serratia	Negative	*Lautropia*	*Dialister*	Negative		*Carnobacterium*	Positive	*Finegoldia*
Acinetobacter	Negative	Lentimicrobiaceae	*Dolosigranulum*	Positive		*Catonella*	Negative	*Roseburia*
		Moraxellaceae	*Filifactor*	Positive		*Comamonas*	Negative	*Staphylococcus*
		Mycoplasma	*Johnsonella*	Negative		*Corynebacterium*	positive	
		Odoribacter	Lachnospiraceae	ND		*Dialister*	Negative	
		Peptococcus	*Lactobacillus*	Positive		*Eikenella*	Negative	
		Rothia	*Lautropia*	Negative		*Escherichia-Shigella*	Negative	
		Staphylococcus	*Moraxella*	Negative		*Flexilinea*	Negative	
		Actinobacillus	*Oceanivirga*	Negative		*Johnsonella*	Negative	
		Actinomarinales	*Olsenella*	Negative		Lentimicrobiaceae	ND	
		Clostridiales	Paludibacteraceae	ND		Moraxella	Negative	
			Parvimonas	Negative		Muribaculaceae	ND	
			Peptococcus	Positive		*Mycoplasma*	ND	
			Propionibacteriaceae	ND		*Oceanivirga*	Negative	
			Rikenellaceae RC9 gut group	ND		*Olsenella*	Positive	
			Roseburia	Positive		Paludibacteraceae	ND	
			Ruminococcaceae UCG-014	ND		*Parvimonas*	Negative	
			Staphylococcus aureus	Positive		*Peptococcus*	Positive	
			Streptobacillus	Negative		*Peptoniphilus*	Positive	
						Rikenellaceae RC9 gut group	ND	
						Roseburia	Positive	
						Ruminococcaceae	ND	
						Serratia	Negative	
						Stenotrophomonas	Negative	
						Streptobacillus	Negative	
						Tannerella	Negative	
						Treponema	Negative	
**14% (7/50) taxa reduced to sub-detectable level**	**71% (5/7) Gram negative**		**45% (24/53) taxa reduced to sub-detectable level**	**50% (12/24) Gram negative**		**53% (32/61) taxa reduced to sub-detectable level**	**59% (19/32) Gram negative**	

Supplementary Table 2: The under 1% RA taxa that were reduced to sub-detectable levels within the 1st two weeks of treatment and those that replaced them within the same period of treatment. ND =Gram stain status not defined.

Supplementary text 3: Change in microbiome in relation to treatment outcome.

Assessment of TB sputum culture status revealed 19% (12/65), 74% (48/65) and 8% (5/65) were culture- negative, positive, and indeterminate (contaminated with no clear TB status) by month two of treatment, respectively. We then asked whether regimen interaction with microbiome affected culture conversion. Patients who converted to negative within eight weeks of treatment (n = 12), showed a consistent decline in taxon richness, and phylogenetic diversity without rebounding (Figure S 2). Those who did not convert (culture positive at week eight (n = 48) had significant reduction in richness and phylogenetic diversity in first two weeks of treatment, ANOVA p<0·001 in both cases but exhibited slight recovery in all diversity indices by month 2 of treatment (figure 8B). The indeterminate cases (contaminated and neither positive nor negative for TB (n = 6) had sharp recovery of alpha diversity

that overtook pre-treatment level by month two of treatment (figure 8C). Taxa evenness remained stable in all the three treatment outcome groups (Supplementary figure S7)·

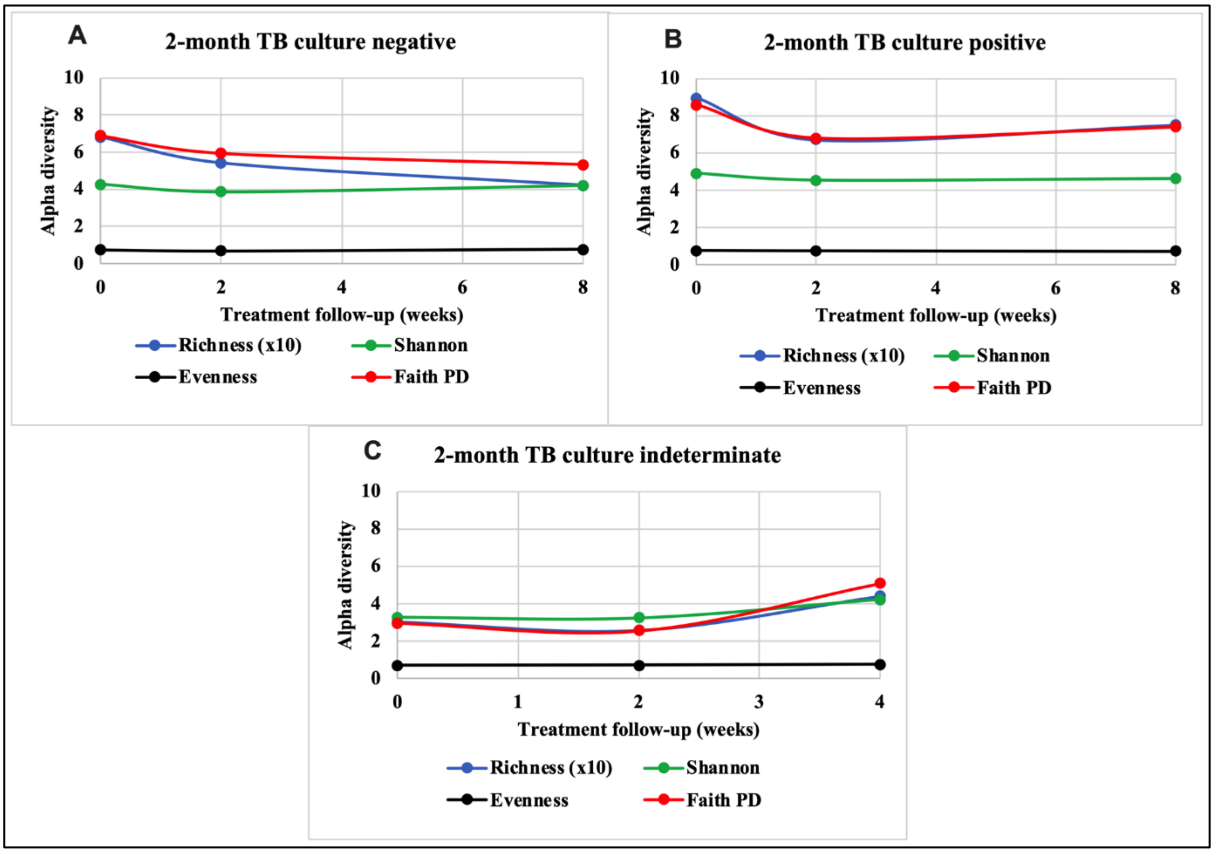

Supplementary figure S7: Relationship of change in microbiome and 2-month TB culture conversion. A) Culture converted with consistent reduction in alpha diversity without recovery. B) Non-culture converted with sharp fall in alpha diversity in 1st 2 weeks of treatment followed by a slight recovery. C) Indeterminate with reduction in richness and phylogenetic diversity by week two of treatment followed by sharp recovery by month 2 of treatment.

References

1· Boeree MJ, Heinrich N, Aarnoutse R, Diacon AH, Dawson R, Rehal S, et al· High-dose rifampicin, moxifloxacin, and SQ109 for treating tuberculosis: a multi-arm, multi-stage randomised controlled trial. Lancet Infect Dis· 2017;17:39–49.

2· Aarnoutse R, Kibiki G, Reither K, Semvua H, Haraka F, Mtabho C, et al· Pharmacokinetics, tolerability, and bacteriological response of rifampin administered at 600, 900, and 1,200 milligrams daily in patients with pulmonary tuberculosis. Antimicrob Agents Chemother. 2017;61:e01054-17.

3· Illumina· Preparing 16S Ribosomal RNA Gene Amplicons for the Illumina MiSeq System· Illumina Tech Note· 2011.
